# Thermal-Stability and Reconstitution Ability of *Listeria* Phages P100 and A511

**DOI:** 10.3389/fmicb.2017.02375

**Published:** 2017-12-05

**Authors:** Hanie Ahmadi, Devon Radford, Andrew M. Kropinski, Loong-Tak Lim, Sampathkumar Balamurugan

**Affiliations:** ^1^Department of Food Science, University of Guelph, Guelph, ON, Canada; ^2^Guelph Research and Development Centre, Agriculture and Agri-Food Canada, Guelph, ON, Canada; ^3^Department of Pathobiology, Ontario Veterinary College, University of Guelph, Guelph, ON, Canada

**Keywords:** Bacteriophage, *Listeria monocytogenes*, thermal-stability, transmission electron microscopy, ready-to eat meat, food safety

## Abstract

The study evaluated the thermal-stability of *Listeria* phages P100 and A511 at temperatures simulating the preparation of ready-to-eat meats. The phage infectivity after heating to 71°C and holding for a minimum of 30 s, before eventually cooling to 4°C were examined. Higher temperatures of 75, 80, and 85°C were also tested to evaluate their effect on phages thermal-stability. This study found that despite minor differences in the amino acid sequences of their structural proteins, the two phages responded differently to high temperatures. P100 activity declined at least 10 log (PFU mL^-1^) with exposure to 71°C (30 s) and falling below the limit of detection (1 log PFU mL^-1^) while, A511 dropped from 10^8^ to 10^5^ PFU mL^-1^. Cooling resulted in partial reconstitution of P100 phage particles to 10^3^ PFU mL^-1^. Exposure to 75°C (30 s) abolished A511 activity (8 log PFU mL^-1^) and both phages showed reconstitution during cooling phase after exposure to 75°C. P100 exhibited reconstitution after treatment at 80°C (30 s), conversely A511 showed no reconstitution activity. Heating P100 to 85°C abolished the reconstitution potential. Substantial differences were found in thermal-stability and reconstitution of the examined phages showing A511 to be more thermo-stable than P100, while P100 exhibited reconstitution during cooling after treatment at 80°C which was absent in A511. The differences in predicted melting temperatures of structural proteins of P100 and A511 were consistent with the observed differences in thermal stability and morphological changes observed with transmission electron microscopy.

## Introduction

*Listeria monocytogenes* continues to be a pathogen of concern in ready-to-eat (RTE) meat products causing listeriosis, a life threatening foodborne disease that primarily affects older adults, pregnant women, newborns, and adults with weakened immune systems. Due to high mortality rate of approximately 25–30%, the disease ranks among the most severe foodborne illnesses (Vazquez-Boland et al., 2001). Worldwide human listeriosis cases have been linked to the consumption of contaminated meats, seafood, delicatessen (deli) meats, dairy and poultry products (Painter, 2007; Centers for Disease Control and Prevention, 2017; European Centre for Disease Prevention and Control, 2017). In Canada, a listeriosis outbreak associated with RTE meat products sickened a total of 57 Canadians and claimed 22 lives (Gilmour et al., 2010).

Food processors are advised to reduce or eliminate *L. monocytogenes* in RTE meat and poultry products by employing antimicrobial agents/processes and/or post-lethality procedure(s) (Canadian Food Inspection Agency, 2011). Antimicrobial agents are food additives which allow no more than 2 log CFU g^-1^ increase in *L. monocytogenes* throughout the stated shelf-life of the product. Chemical antimicrobials such as lactate and diacetate have been approved by Health Canada to control the growth of *L. monocytogenes* in RTE meat and poultry products (Health Canada, 2011).

In the last decade, several alternative processing and preservation technologies have been developed to enhance the safety of food. For example, bacteriophages have been investigated as antimicrobial agents in food systems, including meats, cut fruits, vegetables and milk (Leverentz et al., 2001; Whichard et al., 2003; Tabla et al., 2012; Chibeu et al., 2013; Ahmadi et al., 2015; Murray et al., 2015; Radford et al., 2017). Bacteriophage preparation such as LISTEX^TM^ P100 and LMP-102 are approved by regulatory agencies as processing aids, for use in raw and RTE foods to combat *L. monocytogenes* contamination (U.S. Food and Drug Administration, 2006; Health Canada, 2011). These commercial phage formulations are preservative-free and do not impact sensory and quality attributes of food (Brovko et al., 2012). As a processing aid, the bacteriophage suspension is sprayed on to the surface of products or added by dipping as a liquid prior to packaging (U.S. Food and Drug Administration, 2006). These methods may not be ideal, as they lead to the potential inactivation of the bacteriophages as a result of dilution of phages with other materials such as wash fluids (Anany et al., 2011). This problem may be overcome by incorporating the phage into the raw meat formulation as an additive to ensure phages are applied and distributed uniformly throughout the raw product matrix.

Stability of phages during heat pasteurization and cooking during industrial food production is a particular concern in their application as an additive. Considerable work has been focused on understanding thermal stability of lactic acid bacteria (LAB) phages which are the main cause of fermentation failure in the dairy industry (Wilkowske et al., 1954; Müller-Merbach et al., 2005; Buzrul et al., 2007). Buzrul et al. (2007) treated 10 *Lactococcal* bacteriophages in M17 broth at 72°C for 15 min and 90°C for 5 min. The former treatment resulted in total inactivation of just two phages while the latter inactivated half of the phages. Whitehead and Cox (1936) reported that one strain of lactic *Streptococcus* phage was not stable at 70°C for 30 min, and another strain was destroyed by 50°C for 30 min. While many of the LAB phages survived pasteurization treatments of 63°C for 30 min or 72°C for 15 s (Capra et al., 2004; Mercanti et al., 2012), thermal sensitivity of other phages was observed at 68°C and below (Duda et al., 2009; Brovko et al., 2012; Qiu, 2012) demonstrating the diversity in thermal stability of the phages. However, there is lack of information on thermal stability of phages against *L. monocytogenes*, particularly in the context of industrial heat pasteurization.

Here we examined the thermal stability of two broad host range, lytic *Listeria* phages P100 and A511 at temperatures simulating the preparation of RTE meats, which included heating to 71°C and holding for a minimum of 30 s, before eventually cooling to 4°C (Food Safety and Inspection Service, 1999; Canadian Food Inspection Agency, 2013). TEM analyses of the two *Listeria* phages were performed to understand structural changes under different thermal treatments. Relative estimates of minimum melting temperatures of structural proteins were also determined using Tm (melting temperature) predictor algorithm (Ku et al., 2009) to determine the differences between thermal-stability of P100 and A511 at protein sequence level.

## Materials and Methods

### Establishment of *Listeria* Cultures

*Listeria monocytogenes* strain 08-5578 (serotype 1/2a) obtained from The National Microbiology Laboratory, Canadian Science Centre for Human and Animal Health (Winnipeg, MB, Canada) was used. Overnight cultures of *L. monocytogenes* were prepared by transferring a single colony of *L. monocytogenes* strain 08-5578 to 5.0 mL TSB (BD Diagnostics, San Jose, CA, United States) in a 15 mL screw cap, sterile Falcon tubes (Fisherbrand, Fisher Scientific International, Inc., Pittsburgh, PA, United States) in a rotary shaker and incubated for 18 h at 37°C and 120 rpm.

### Bacteriophage Preparation

*Listeria* phage P100 was obtained from MICREOS Food safety, Inc. (Netherlands) in PBS at concentrations of 10^10^–10^11^ PFU mL^-1^. *Listeria* phage A511 was obtained from The Félix d’Hérelle Reference Center for Bacterial Viruses, University of Laval (Quebec, QC, Canada). Phage A511 was propagated as described by Radford et al. (2016) with some minor modifications. Briefly, 200 μL of *L. monocytogenes* subculture (10^9^ CFU mL^-1^) and 100 μL of phage A511 (10^9^ PFU mL^-1^) were added to 4 mL of top agar supplemented with CaCl_2_ (TSB, 0.5% agar, 10 mM CaCl_2_). The solution was uniformly mixed and poured onto sterile TSA plates (Fisherbrand). Plates were incubated at 30°C for 18 h to form a top agar layer of phage-host co-culture. After incubation, 5 mL of SM buffer (5.8 g NaCl, 2 g MgSO_4_.7H_2_O, 50 mL Tris-Cl at pH 7.5, 0.1 g gelatin) was added to the plates to cover the surface of top agar entirely and refrigerated at 4°C overnight. After refrigeration all the liquids were extracted using a micropipette and filtered through 0.22 μm membranes. The filtrate was retained and stored at 4°C until use (Radford et al., 2016). The titre of propagated phages was determined by adding serial dilutions to agar overlays and incubating as previously described by Kropinski et al. (2009).

### Thermal Stability of P100 and A511 at Constant Temperature

The heating vessels used were standard 160 mL screw cap glass dilution bottles. Each bottle contained 90 mL of SM buffer (pH 7.5) with a magnetic stir bar. Prior to inoculation, SM buffer was equilibrated to the test temperature of 45, 55, or 65°C in a circulating water bath (TW-2.03, Rose Scientific, Edmonton, AB, Canada). In the case of all experiments described in this manuscript the treatment temperatures were maintained within ± 0.5°C. During heating, the water level was maintained at least 2.0 cm above the level of the treatment solution at all times and the magnetic stirrer operated using a MS-01 four position magnetic stirrer (Rose Scientific). Phage P100 (commercially preparation) at 10^10-11^ PFU mL^-1^ and A511 at 10^9^ PFU mL^-1^ (highest titre obtained by the propagation method indicated in the previous section) were used in the thermal stability experiment. An aliquot of 10 mL of P100 or A511 at 4°C was added to the heating solution and temperature monitored till the heating solution reached the respective treatment temperatures following which 1.0 mL sample was taken at *t* = 0 and then every 15 min for 75 min. Due to instability of phages in the 65°C treatment, samples were taken every 2.5 min for 20 min to assess thermal stability. Samples were immediately diluted in SM buffer and the phage titers determined with the phage overlay assay (Kropinski et al., 2009). All experiments were performed in triplicate and each sample plated in duplicate. A linear regression curve of the phage PFU mL^-1^ against time was constructed. Best fit regression lines for each plot were used to calculate the decimal reduction time (*D*-value; time required for one log reduction in plaque numbers at a given temperature).

### Thermal Stability of P100 and A511 during Heating-Holding-Cooling Treatments

Treatments involved heating the phage suspensions from 4°C to 71, 75, 80, or 85°C, (referred to as heating) and holding for 30 s (referred to as holding) and then cooling back to 4°C (referred to as cooling) to check the stability of phages. The heating vessels used were standard 160 mL screw cap glass dilution bottles. Each bottle contained 90 mL of SM buffer (pH 7.5) with a magnetic stir bar. Prior to inoculation, the heating medium was equilibrated to 4°C. Then, 10 mL of P100 (10^11^ PFU mL^-1^) or A511 (10^9^ PFU mL^-1^) at 4°C was added to the treatment solution and placed in a heated circulating water bath set at 72°C (temperature of the water bath was set to 1°C greater than the desired peak heating temperatures) and the temperature of the treatment solution monitored using a digital thermometer (Traceable Calibration Control, VWR International, Radnor, PA, United States). Aliquot of 1.0 mL samples were taken at *t* = 0 (4°C), 40°C and then at every 10°C increase in sample temperature from 40 to 70°C, and 71°C (heating). The heating vessel was then held at 71°C for 30 s (holding) and another sample was taken. Immediately after heating and holding, the heating vessel was transferred to circulating cooling water bath, cooled using a glycol circulating cooling jacket set at -3°C. Aliquots of 1.0 mL samples were taken at every 10°C reduction in sample temperature, until the treatment solution temperature reached 4°C (cooling). Samples were immediately diluted in SM buffer and the phage titers determined with the phage overlay assay (Kropinski et al., 2009). Based on preliminary results obtained, similar experiments were repeated at 75, 80, and 85°C peak heating temperatures for P100 while 75 and 80°C for phage A511. Time required for samples to reach peak temperature of 71, 75, 80, and 85°C were monitored and were consistently around 477 ± 14, 519 ± 38, 597 ± 74, and 711 ± 41 s respectively. Cooling time from peak temperatures to 4°C was approximately 1169 ± 46 s. P100 with initial concentration of 10^5^ and 10^8^ (PFU mL^-1^) in treatment solutions were also examined at peak heating (71°C)-holding-cooling treatment and A511 with initial concentration of 10^5^ PFU mL^-1^ in treatment solution was investigated at peak heating (75°C)-holding-cooling treatment. All experiments were performed in triplicate and each sample plated in duplicate.

### Preparation and Visualization of Treated Phage

Transmission electron microscopy was performed on samples to visualize the effect of heat on phage morphology. Phage suspensions were concentrated by density gradient centrifugation (Cardarelli et al., 2010). The concentrated phages were diluted 1:20 with double–distilled water and directly negative stained for TEM. Copper–rhodium (400-mesh) grids were covered with a thin layer of amorphous carbon made hydrophilic by 45 s vacuum glow-discharge. Phage solution samples (4–6 μL) were placed on individual grids and left for 2 min to adsorb onto the carbon. Excess sample was gently removed by touching filter paper to the edge of the droplet after adsorption was stablished. Excess small molecule contaminants were washed from the grid with three rinses of water. Sample on the grids were stained with 2% (w/v) uranyl acetate (Cardarelli et al., 2010). A Tecnai G2 transmission electron microscope (FEI Company, Hillsboro, OR, United States) at Electron Microscopy Unit, University of Guelph (Guelph, ON, Canada) was used for visualization, operating at 200 kV under variable magnification. Images were acquired using the Gatan Ultascan 4K CCD (Pleasanton, CA, United States) and Gatan Digital Micrograph software imaging system.

### Statistical Analysis

All experiments were carried out in triplicate and each sample plated in duplicate. Statistically analysis and analysis of variance (ANOVA) were conducted using the SAS Statistic Package (SAS Institute Inc., Cary, NC, United States), at a confidence interval of 95% to examine the variance in titre of the phage P100 and A511 at temperature points and between treatments. In cases where statistical differences between means were detected, Tukey’s test was applied for multiple pair-wise comparisons between treatment means.

## Results and Discussion

### Determination of Thermal Stability at Constant Temperature Treatments

Thermal stability of *Listeria* phages were examined at constant temperatures and heating-holding-cooling treatments. In order to estimate the thermal stability of P100 and A511 at constant temperatures, both phages underwent heat treatments of 45, 55, and 65°C. P100 and A511 are well characterized and their genes sequenced (Carlton et al., 2005; Klumpp et al., 2008). These phages both are members of the *Myoviridae* family of bacteriophages, with small differences (3 genes) in their tail interfaces. While highly similar at the primary sequence level, these phages exhibited different thermal responses. P100 and A511 were stable at 45°C for 75 min (data not shown). However, unlike A511, P100 exhibited thermal sensitivity at 55°C and lost ∼2 log PFU mL^-1^ activity. At the end of 20 min heat treatment at 65°C, A511 activity was reduced by 1 log cycle (PFU mL^-1^), while P100 was more sensitive with decreasing 4 log cycles in activity after 20 min (data are not shown). This is a significant (*P* < 0.05) decrease in *D*-value with the increase in treatment temperatures (**Table 1**). Increasing temperature to 65°C significantly (*P* < 0.05) decreased the *D*-values to 6.03 ± 0.47 and 16.80 ± 2.84 min for P100 and A511, respectively. Yamagishi and Ozeki (1972) suggested that thermal-sensitivity of phages may depend on their DNA content. A511 features a slightly smaller head diameter (87.36 nm) than P100 (89.55 nm). This feature along with the larger DNA molecule in the A511 head, with a unit genome 3.1 Kb larger than that of P100 (131,384 bp versus 134,494 bp) (Klumpp et al., 2008), indicating a denser head capsid content, which could explain the observed higher stability of A511.

**Table 1:** Thermal-stability (expressed as *D*-values in min) for P100 and A511 at 45, 55, and 65°C in SM buffer.

Phage	Temperature (°C)	*D*-value (min)	*Y*	*R*^2^
P100	45	909.09 ± 174.09^a^	Y = -0.0011x + 10.394	0.945
	55	42.01 ± 1.45^b^	Y = -0.0238x + 9.8568	0.924
	65	6.03 ± 0.47^c^	Y = -0.0595x + 6.7825	0.948
A511	45	10,000.00 ± 5,581.05^d^	Y = -0.0001x + 8.14	0.064
	55	312.50 ± 53.30^e^	Y = -0.0032x + 7.2086	0.993
	65	16.80 ± 2.84^f^	Y = -0.1659x + 4.9238	0.915

*Different superscript letters associated with the numbers in the columns indicate statistical significance between two mean. Level of significance was 0.05.*

The coefficient of determination *R*^2^ was > 0.90 in all cases, indicating the data fits well with the linear regression model, except for A511 at 45°C (**Table 1**). At 45°C, A511 shows effectively no decline in titre, hence the near zero regression slope, and sensitivity to technical variation in measurements. Simple logarithmic inactivation of phages (Pollard and Reaume, 1951), as well as non-linear relationship inactivation model for thermal-resistant phages (a mixture of two virus population which are similar but have more different thermal-stability) has been previously reported (Wilkowske et al., 1954; Quiberoni et al., 2003). Linearity in P100 and A511 regression model affirmed rather homogenous population by virtue of thermal-stability in phage preparation. Unlike dairy phages which have been reported to be stable to 63°C (Quiberoni et al., 2003; Ebrecht et al., 2010; Chen et al., 2017), P100 and A511 exhibited sensitivity to heat and instability at 65°C (**Table 1**). Although both phages infect the same host species, they show significant differences in their thermal-stability. It suggested that the major lethal event during heat treatments was due to the release of DNA from the phage particles (Yamagishi et al., 1973; Atamer et al., 2010; Guglielmotti et al., 2012).

### Determination of Thermal Stability at Heating-Holding-Cooling Treatments

**FIGURE 1:**
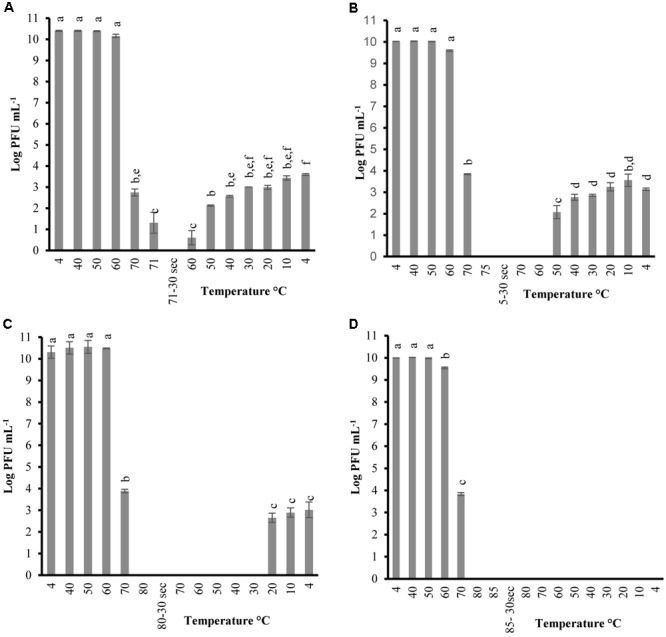
Changes in phage P100 infectivity (in log PFU mL^-1^) following the heating-holding-cooling trial in SM buffer at peak heating temperatures of 71°C **(A)**; 75°C **(B)**; 80°C **(C)**; 85°C **(D)**. Thirty seconds on the *x*-axis refers to holding time of 30 s at pertaining temperatures. Different superscript letters associated with the numbers on the bars indicate statistical significance between two means with the level of significance of 0.05. Error bars denote standard deviation.

**FIGURE 2:**
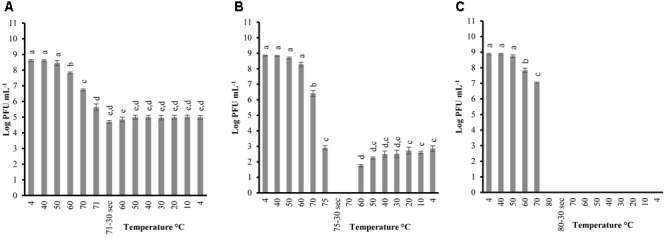
Changes in phage A511 infectivity (in log PFU mL^-1^) following the heating-holding-cooling trial in SM buffer at peak heating temperatures of 71°C **(A)**; 75°C **(B)**; 80°C **(C)**. Thirty seconds on the *x*-axis refers to holding time of 30 s at pertaining temperatures. Different superscript letters associated with the numbers on the bars indicate statistical significance between two means with the level of significance of 0.05. Error bars denote standard deviation.

In order to determine the thermal-stability of *Listeria* phages at temperatures simulating the preparation of RTE meats, P100 and A511 were treated by heating to 71°C-holding 30 s-cooling to 4°C. **Figures 1A, 2A** show decline in P100 and A511 activity with increasing temperature during the heating phase. P100 reached below the limit of detection (1 log PFU mL^-1^) after 30 s of exposure to 71°C. When the same treatment was applied to A511, phage activity declined by only 3 log cycles (PFU mL^-1^), and retained activity even after 30 s of holding at 71°C, indicating that in spite of similar primary sequence of their structural proteins, A511 is more heat stable than P100. Release of phage DNA from viral capsids, as well as decomposition of the phages into head and tail structure and tail aggregation are known as common mechanisms observed for viruses thermal inactivation.

Unlike previous studies examining the effect of heat on LAB and enterobacterial phages (Kawai, 1999; Buzrul et al., 2007; Qiu, 2012), P100 exhibited partial reconstitution (3–4 log PFU mL^-1^ of activity) after heating (71°C, holding 30 s) followed by cooling to 4 ± 0.5°C (**Figure 1A**). Hereafter, the term reconstitution refers to the phenomenon when phage infectivity/activity reaches below the limit of detection during heating and/or holding, while exhibiting renewed infectivity during cooling phase. Since A511 did not exhibit a loss of the complete activity (below limit of detection) at 71°C, thermal-stability was tested to 75°C, a temperature which renders A511 inactive (**Figure 1A**), in order to examine if phage particles are capable of reconstitution. In addition, this approach enabled us to also examine if the phenomenon of reconstitution was observed in P100 at temperatures higher than 71°C. Raising the maximum temperature to 75°C yielded complete inactivation of P100 (**Figure 1B**), while complete inactivation of A511 was observed after 30 s of holding (**Figure 2B**). Cooling to 4.0°C after heating to 75°C resulted in partial reconstitution for both phages (**Figures 1B, 2B**). This reconstitution activity was not observed when A511 was exposed to 80°C (**Figure 2C**), yet P100 particles reconstituted after 30 s of exposure to 80°C (**Figure 1C**). Despite the similarity of the two phages, A511 exhibited more thermo-stability compared to P100, but failed to show the same level of reconstitution. Raising the temperature to 85°C abolished the reconstitution ability for P100 (**Figure 1D**). Considering the thermal inactivation and reconstitution of these phages, it could be speculated that although A511 had higher thermal stability during the heating stage compared to P100 (**Figures 1, 2**), lack of reconstitution in A511 during the cooling stage compared to P100 suggests that heating of A511 results in an irreversible damage to the phage particle compared to P100 where the damage was reversible.

**Table 2** shows the temperature and phage population when reconstitution was observed, as well as phage population at the end of the heating-holding-cooling trial (4°C). There were an inverse relationship between peak heating temperature and the temperature at which reconstitution was observed. With an increase in peak heating temperature from 71 to 75 or 80°C, reconstitution was observed at 60, 50, and 20°C, respectively. The reconstitution of *Listeria* phages significantly (*P* < 0.05) decreased with an increase of temperature. P100 showed reduced reconstitution from 80°C (3.01 ± 0.36 log PFU mL^-1^) compared to lower peak temperature of 71°C (3.59 ± 0.04 log PFU mL^-1^). Similarly, A511 reconstitution of 2.84 ± 0.18 log PFU mL^-1^ was observed at 75°C, while no phage was detected after cooling following a peak heating temperature of 80°C (**Table 2**). In treatments with higher peak heating temperatures, cooling to lower temperatures was required to detect reconstitution (**Table 2**). It is possible that higher peak heating temperatures could result in irreversible damage/denaturation of the phage particles. This was evident from our results when phage suspension treated to higher peak temperatures, required cooling to lower temperatures to demonstrate the reconstitution phenomenon.

**Table 2:** Reconstitution temperature (°C) and population (Log PFU mL^-1^) of P100 and A511 in heating-holding-cooling trials at different peak temperature treatments.

Bacteriophage	Heating peak temperature (°C)	Reconstitution		
		Temperature^∗^(°C)	Population^∗∗^ (Log PFU ml^-1^)	Population^∗∗∗^ (Log PFU ml^-1^)
P100	71	60	0.60 ± 0.30	3.59 ± 0.04^a^
	75	50	2.07 ± 0.30	3.13 ± 0.05^b^
	80	20	2.65 ± 0.21	3.01 ± 0.36^b^
	85	–	–	–
A511	71	–	–	4.98 ± 0.11
	75	60	1.75 ± 0.07	2.84 ± 0.18
	80	No reconstitution	–	–

*Different superscript letters associated with the numbers in the columns indicate statistical significance between two mean. The level of significance was 0.05. ^∗^Temperature at which reconstitution was observed; ^∗∗^Population when reconstitution was observed; ^∗∗∗^Population at the end of heating-holding-cooling trails.*

Viral inactivation by thermal treatment has been previously studied (Wilkowske et al., 1954; Caldentey et al., 1993; Moroni et al., 2002; Müller-Merbach et al., 2005; Buzrul et al., 2007; Atamer et al., 2013; Jurczak-Kurek et al., 2016; Chen et al., 2017), yet reconstitution during cooling phase after heating treatment has not been widely investigated. Zairi et al. (2014) investigated the effects of heating and cooling cycle (60°C and cooled to 10°C) on gp12 capsid fiber of *Bacillus* phage SPP1 in a buffer solution [500 mm NaCl and 50 mm Na_2_HPO_4_ (pH 8.0)] with a Fluorescence-based Thermal Shift Assay. They reported a loss of protein gp12 capsid fiber structure between 30 and 45°C and unfolding of polypeptide chains of gp12 was observed between 45 and 80°C. However, fast (<1 min) or progressive cooling of the sample back to 10°C led to complete reconstitution of the secondary, tertiary, and quaternary protein structures identical to that of the untreated proteins. Phenomenon of reconstitution for T4 phage tail particles previously was reported by Jayaraman et al. (1997). They observed the short tail fibers of bacteriophage T4 which are composed of gp12 was able to reconstitute after heating to 75°C. We believe that the underlying mechanisms involved phage reconstitution might be similar. Their results are consistent with the observations in this research. Although these researchers examined the capsid fiber protein of phage SPP1 and tail fiber protein of phage T4, our experiment was conducted with whole phage particles and focused on *Listeria* phages relevant to food protection. To date, no reconstitution of *Listeria* phages undergoing heating process has been reported. According to these results, it is possible to conclude that the P100 and A511 proteins have characteristic to retain a native folded state when exposed to denaturing conditions to some extent of temperature elevation. It was more evident for tail structural proteins, especially for P100. The irreversible inactivation of phages occurred at temperatures >80°C due to irreversible unfolding and dissociation of structural proteins.

**FIGURE 3:**
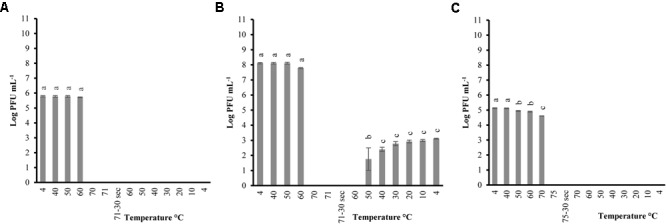
Changes in phage P100 infectivity (in log PFU mL^-1^) following the heating 71°C-holding-cooling trial in SM buffer with initial concentration of 10^5^ PFU mL^-1^ **(A)**; 10^8^ PFU mL^-1^ **(B)**; and phage A511 population following the heating 75°C-holding-cooling trial in SM buffer with initial concentration of 10^5^ PFU mL^-1^ **(C)**. Thirty seconds on the *x*-axis refers to holding time of 30 s at pertaining temperatures Different superscript letters associated with the numbers on the bars indicate statistical significance between two means with the level of significance of 0.05. Error bars denote standard deviation.

Phages were tested with the same heating-holding-cooling protocol at different concentrations to evaluate the reconstitution activity. The lowest peak heating temperature at which complete inactivation and reconstitution was observed was picked to determine any correlation between phage concentrations and thermal-stability. Therefore, peak heating temperatures of 71°C and 75°C was used for P100 and A511, respectively. When P100 with initial concentrations of 10^5^ and 10^8^ (PFU mL^-1^) were examined at heating 71°C-holding-cooling, phages could no longer be detected at 70°C (**Figures 3A,B**), while phage particles were still detected (10^2-3^ PFU mL^-1^) at 70°C during the same treatment when initial concentration of phage was 10^10^ PFU mL^-1^ (**Figure 1A**). Reconstitution of P100 (10^8^ PFU mL^-1^) was observed during cooling starting at 50°C (**Figure 3B**), while the same treatment with initial concentration of 10^10^ PFU mL^-1^ resulted in reconstitution during cooling starting at 60°C (**Figure 1A**). P100 with initial concentration of 10^5^ PFU mL^-1^ exhibited no reconstitution of phage particles with the same treatment (**Figure 3A**). When phage A511 with 10^5^ PFU mL^-1^ were examined at heating 75°C-holding-cooling, no reconstitution activity were observed (**Figure 3C**), while, partial reconstitution was observed with higher initial concentration of 10^8^ PFU mL^-1^ (**Figure 2A**). It is possible that the lower reconstitution ability and thermal stability observed in less concentrated phage solutions might be due to a dilution effect on phage population.

### TEM Analysis on P100 and A511 Structure

**FIGURE 4:**
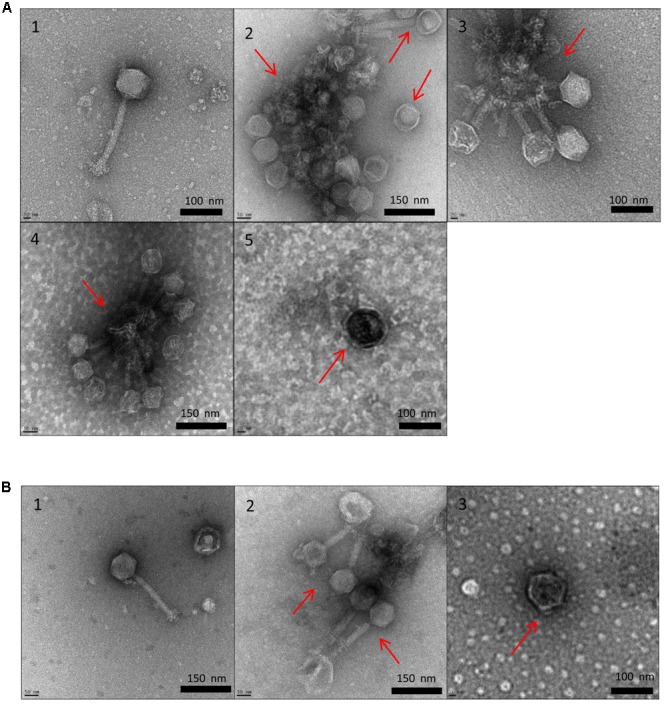
Transmission electron micrographs of *Listeria* phages P100 **(A)** and A511 **(B)** exposed to different treatments. Untreated control (1); heating to 71°C (2); heating to 75°C (3); heating to 80°C (4); heating to 85°C (5).

Transmission electron microscopy allowed us to visualize the physical effects of high temperature on P100 and A511. **Figures 4A1,B1** show untreated controls of P100 and A511, respectively, which feature long, contractile tails and isometric capsids that are characteristic of *Myoviridae* family in the order *Caudovirales* (Zink and Loessner, 1992). Heating P100 to 71°C (**Figure 4A2**) resulted in a mixed population of fully disrupted virions, aggregated tail (Supplementary Figure 2), empty head (dark color), and visually intact phages (white color indicating capsid containing nucleic acid) (**Figure 4A1**). Similar observations were made in treatment to 75 and 80°C (**Figures 4A3,A4**) with tail fibers and baseplates aggregation (Supplementary Figure 3). The tendency to clustering of empty head and aggregated phages particles of the P008 viral particles as a result of exposure to 70°C for 10 min was reported by Atamer et al. (2010). Exposure of P100 to 85°C (**Figure 4A5**) showed only dark isometric capsid structure indicating that they were empty of DNA. TEM images of phage A511 from heating to 75°C displayed less tail aggregation with more visually intact phages (**Figure 4B2**) suggesting higher thermal stability which is in agreement with results presented in **Table 1** and **Figure 2**. Samples treated to 80°C exhibited empty phage capsid without tails similar to that observed for P100 heated to 85°C (**Figure 4B3**).

Transmission electron microscopy observations suggested that tail component of the phage were more thermosensitive and denatured at lower temperatures compared to capsid. Similar observation were made by Qiu (2012) who reported that λ phage tail disrupted at 68°C, while capsid proteins disrupted later at 87°C. Unlike P100, heat-induced damages in A511 was more pronounced after heating to 80°C, while similar damages in P100 were observed when heated to 85°C. This explains the lack of observed reconstitution of A511 when heated to 85°C, while P100 heated to 80°C showed reconstitution (**Figures 1, 2**).

### Predicted Melting Temperature for Structural Proteins

In order to compare the differences between thermal-stability of P100 and A511 at the protein sequence level, relative estimate of minimum melting temperatures of structural proteins were determined using Tm predictor algorithm (Ku et al., 2009). Phage stability and functionality depends on both polar and hydrophobic interactions in their proteins. These interactions have different melting temperatures (Gorania et al., 2010), depending on the specific amino acids and geometry involved in formation of these interfaces. Predicting melting temperature (Tm, the temperature at which 50% of the protein is unfolded) from protein sequences can help with better understanding the molecular basis of thermal stability of proteins. The Tm predictor software determines the melting temperatures using primary sequence and correlative extrapolation of dipeptides interactions based on large representative dataset of thermally characterized proteins (Ku et al., 2009).

When comparing the primary sequence level between P100 and A511, 22 structural protein pairs were predicted as present in both phages. Minimum melting temperatures of these structural proteins were estimated using the Tm predictor and are presented in **Table 3**. The P100 putative head-tail connector, predicted tail components such as tail fiber proteins, tail tip-collar, tail initiator, host recognition protein and baseplate hub, all were predicted to have lower melting temperatures than A511. The result is consistent with our observation of tail aggregation of P100 when exposed to heat. Portal proteins of A511 were predicted to melt at lower temperatures than P100 portal. Therefore, it is likely that heating A511 to 80°C caused the damage to the portal complex, disintegration of phage into head and tail and leakage of DNA (irreversible), while P100 retained the portal complex when exposed to 80°C (**Table 3**). This might be a plausible source of difference in ability of P100 and A511 to reconstitution after heating to 80°C and during cooling to 4°C.

**Table 3:** Comparison between melting temperature of P100 and A511 proteins based on Tm predictor.

Structural Functional group	P100		A511		Descriptions (P100 relative to A511)
	Protein	Melting class (°C)	Protein	Melting class (°C)	
Putative head-tail connector	gp18	>65	gp87	>65	• 20aa shorter than A511
					• Lower melting point than A511
					• Plausible source of difference in tail disintegration
Predicted tail component	gp19	∼58	gp88	∼59	• Lower melting point than A511
protein related to side fibers					• Plausible source of difference in tail aggregation
Hub and entry hydrolase	gp29	55–65	gp97	>65	• Lower melting point than A511
					• Plausible source of difference in tail aggregation, and tail disintegration
Putative tail fiber protein	gp30	∼59	gp99	∼60	• Lower melting point than A511
					• Plausible source of difference in tail aggregation
Predicted baseplate	gp32	55–65	gp106	∼60	• Lower melting point than A511
component					• Plausible source of difference in tail aggregation
Receptor binding/fiber	gp39	55–65	gp108	55–60	• Lower melting point than A511
protein					• 67aa shorter than A511
					• Plausible source of difference in tail aggregation
Portal protein	gp14	55–60	gp83	∼55	• Higher melting point than A511
					• Plausible source of difference in tail aggregation

## Conclusion

The study evaluated the thermal-stability of *Listeria* phages P100 and A511 at temperatures simulating the preparation of RTE meats. P100 and A511 are very similar but not identical (98% identical over 95% of A511 genome), and they did not follow the same trend in thermal-stability and phage particle reconstitution, indicating thermal-stability is phage particle dependent. P100 showed more sensitivity to heat, being fully inactivated after exposure to 71°C and holding 30 s, yet was able to reconstitute even from heating to 80°C. However, phage A511 showed less sensitivity to heat and was not fully inactivated by exposure 71°C but didn’t show reconstitution after heating to 80°C. TEM visualization showed the detrimental effect of heat on phage structure causing tail aggregation, detachment of phage head and tail and generation of empty capsid at higher temperature. While there are many similarities between melting temperature of functional proteins in P100 and A511, there are specific differences as well, notably among structural proteins based on Tm prediction, which would explain the plausible source of difference in ability of P100 compared to A511 in reconstitution after heating to 80°C and during cooling to 4°C. Further analysis by nuclear magnetic resonance spectroscopy (NMR) or circular dichroism (CD) measurements for protein structure determination and folding and unfolding of proteins would help to understand these differences. Evaluation of the thermal stability of these phages in food matrix and their efficacy to control *L. monocytogenes* is required to determine if phages can be used as an additive in foods.

## Author Contributions

HA performed all of the bacterial and phage assays presented, phage propagation, sampling, and data analysis. HA and DR both performed transmission electron microscopy and interpreted the resulting images. DR analyzed and interpreted the predicted protein melting temperature data. HA, DR, AK, SB, and L-TL contributed to experimental design for all assays presented. HA wrote the manuscript with input from SB, AK, DR, and L-TL. SB is the principle investigator of the project who was responsible for preparation of project proposal, procure funding, resource allocation and along with the AK and L-TL provided overall guidance and mentorship throughout the scope of this project.

## Conflict of Interest Statement

The authors declare that the research was conducted in the absence of any commercial or financial relationships that could be construed as a potential conflict of interest. The reviewer GW and handling Editor declared their shared affiliation.

## Abbreviations

CFU: colony forming units
PBS: phosphate-buffered saline
PFU: plaque forming units
SM: Saline-Magnesium buffer
TEM: transmission electron microscopy
TSA: tryptic soy agar
TSB: tryptic soy broth
