# Supplementary material for: Thermal-Stability and Reconstitution Ability of Listeria Phages P100 and A511
Source: Front Microbiol. 2017 Dec 5;8:2375. doi: 10.3389/fmicb.2017.02375 (PMC5723416; doi:10.3389/fmicb.2017.02375)
Supplement: Supplementary file 1 [file Image_1.pdf]

Supplementary Figure 1

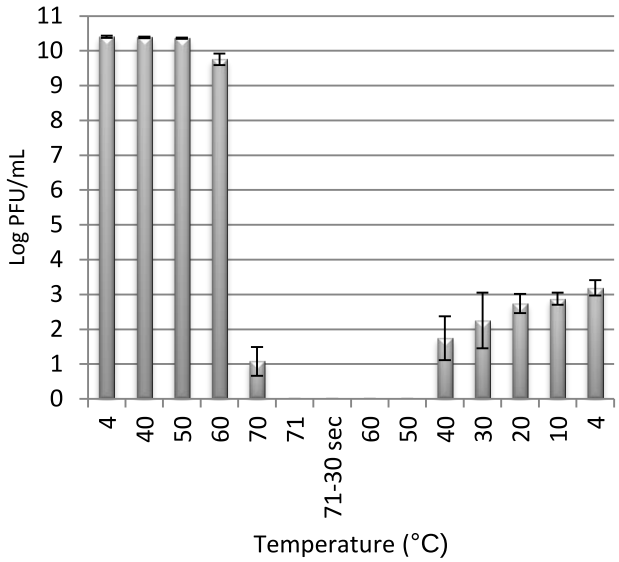

Supplementary Figure 1. Changes in P100 infectivity (in log PFU/mL) following the heating-holding-cooling trial in Meat juice medium at peack heating temperature of 71 °C.
