# Supplementary material for: Thermal-Stability and Reconstitution Ability of Listeria Phages P100 and A511
Source: Front Microbiol. 2017 Dec 5;8:2375. doi: 10.3389/fmicb.2017.02375 (PMC5723416; doi:10.3389/fmicb.2017.02375)

Supplementary Figure 2. Aggregation of P100 phage tail elements when heated to 71°C. TEM magnification is x29000.

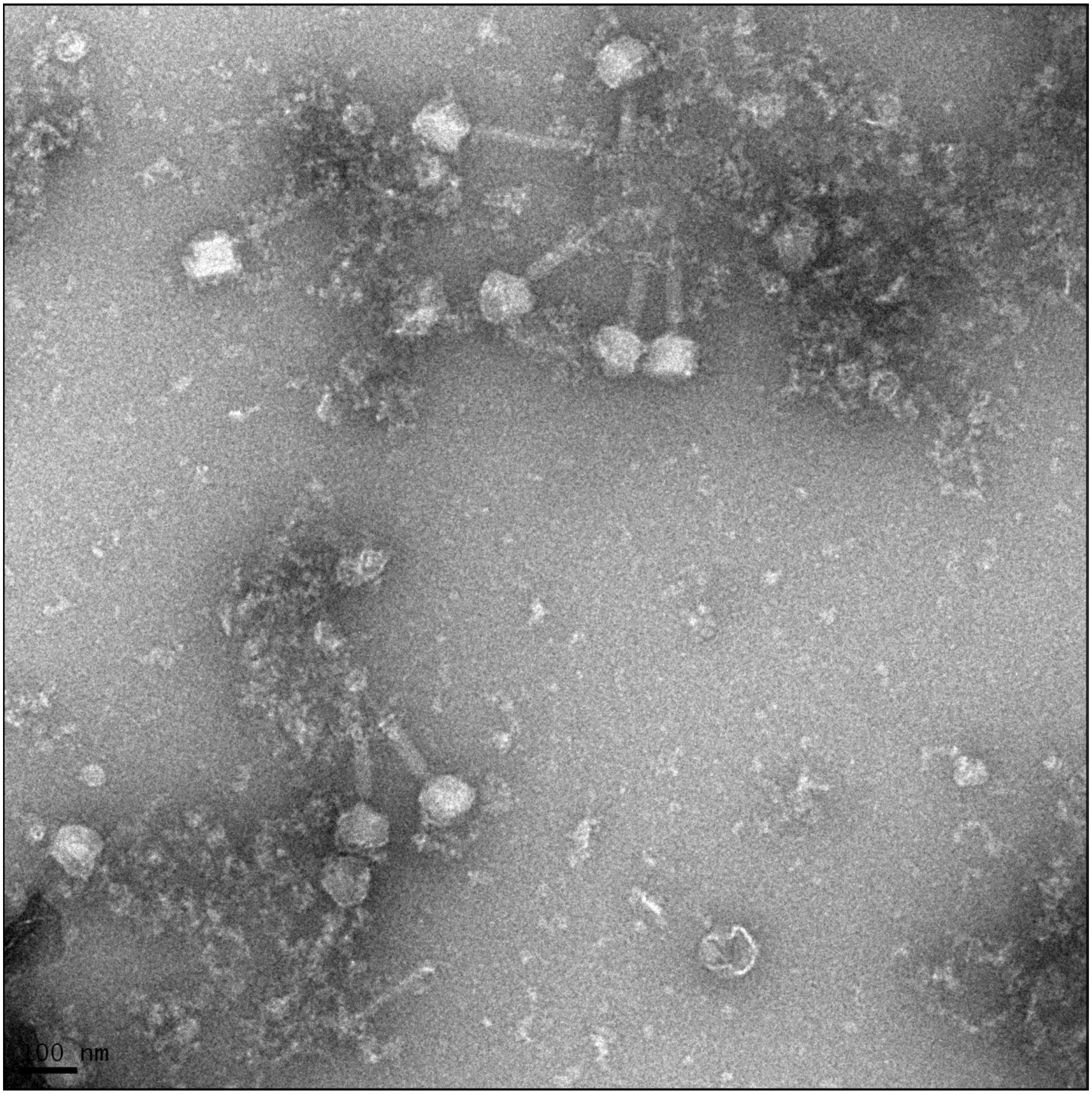

Supplement: Supplementary file 2 [file Image_2.pdf]
